# Silver Sulfadiazine Eradicates Antibiotic-Tolerant *Staphylococcus aureus* and *Pseudomonas aeruginosa* Biofilms in Patients with Infected Diabetic Foot Ulcers

**DOI:** 10.3390/jcm9123807

**Published:** 2020-11-25

**Authors:** Enea Gino Di Domenico, Barbara De Angelis, Ilaria Cavallo, Francesca Sivori, Fabrizio Orlandi, Margarida Fernandes Lopes Morais D’Autilio, Chiara Di Segni, Pietro Gentile, Maria Giovanna Scioli, Augusto Orlandi, Giovanna D’Agosto, Elisabetta Trento, Daniela Kovacs, Giorgia Cardinali, Annunziata Stefanile, Tatiana Koudriavtseva, Grazia Prignano, Fulvia Pimpinelli, Ilaria Lesnoni La Parola, Luigi Toma, Valerio Cervelli, Fabrizio Ensoli

**Affiliations:** 1 Microbiology and Virology, San Gallicano Dermatological Institute IRCCS, 00144 Rome, Italy; ilaria.cavallo@ifo.gov.it (I.C.); effe.sivori@gmail.com (F.S.); giovanna.dagosto@ifo.gov.it (G.D.); elisabetta.trento@ifo.gov.it (E.T.); grazia.prignano@ifo.gov.it (G.P.); fulvia.pimpinelli@ifo.gov.it (F.P.); fabrizio.ensoli@ifo.gov.it (F.E.); 2 Department of Plastic and Reconstructive Surgery, University of Rome Tor Vergata, 00144 Rome, Italy; bdeangelisdoc@gmail.com (B.D.A.); fabrizio.orlandi86@gmail.com (F.O.); mflmdautilio@gmail.com (M.F.L.M.D.); ladis.disegni@gmail.com (C.D.S.); pietrogentile2004@libero.it (P.G.); valeriocervelli@virgilio.it (V.C.); 3 Department of Anatomic Pathology, University of Rome Tor Vergata, 00144 Rome, Italy; scioli07@hotmail.it (M.G.S.); orlandi@uniroma2.it (A.O.); 4 Cutaneous Physiopathology, San Gallicano Dermatological Institute, IRCCS, 00144 Rome, Italy; daniela.kovacs@ifo.gov.it (D.K.); giorgia.cardinali@ifo.gov.it (G.C.); 5 Department of Clinical Experimental Oncology, IRCCS Regina Elena National Cancer Institute, 00144 Rome, Italy; stefanile.nunzia@gmail.com (A.S.); tatiana.koudriavtseva@ifo.gov.it (T.K.); 6 Lichen Sclerosus Unit, Department of Dermatology, STI, Environmental Health, Tropical and Immigration, San Gallicano Dermatological Institute, IRCCS, 00144 Rome, Italy; ilaria.lesnoni@ifo.gov.it; 7 Department of Research, Advanced Diagnostics, and Technological Innovation, Translational Research Area, IRCCS Regina Elena National Cancer Institute, 00144 Rome, Italy; luigi.toma@ifo.gov.it

**Keywords:** biofilm, diabetic foot ulcer, chronic wound, silver sulfadiazine, *Staphylococcus aureus*, *Pseudomonas aeruginosa*

## Abstract

Infections are among the most frequent and challenging events in diabetic foot ulcers (DFUs). Pathogenic bacteria growing in biofilms within host tissue are highly tolerant to environmental and chemical agents, including antibiotics. The present study was aimed at assessing the use of silver sulfadiazine (SSD) for wound healing and infection control in 16 patients with DFUs harboring biofilm-growing *Staphylococcus aureus* and *Pseudomonas aeruginosa*. All patients received a treatment based on a dressing protocol including disinfection, cleansing, application of SSD, and application of nonadherent gauze, followed by sterile gauze and tibio-breech bandage, in preparation for toilet surgery after 30 days of treatment. Clinical parameters were analyzed by the T.I.M.E. classification system. In addition, the activity of SSD against biofilm-growing *S. aureus* and *P. aeruginosa* isolates was assessed in vitro. A total of 16 patients with *S. aureus* and *P. aeruginosa* infected DFUs were included in the study. Clinical data showed a statistically significant (*p* < 0.002) improvement of patients’ DFUs after 30 days of treatment with SSD with significant amelioration of all the parameters analyzed. Notably, after 30 days of treatment, resolution of infection was observed in all DFUs. In vitro analysis showed that both *S. aureus* and *P. aeruginosa* isolates developed complex and highly structured biofilms. Antibiotic susceptibility profiles indicated that biofilm cultures were significantly (*p* ≤ 0.002) more tolerant to all tested antimicrobials than their planktonic counterparts. However, SSD was found to be effective against fully developed biofilms of both *S. aureus* and *P. aeruginosa* at concentrations below those normally used in clinical preparations (10 mg/mL). These results strongly suggest that the topical administration of SSD may represent an effective alternative to conventional antibiotics for the successful treatment of DFUs infected by biofilm-growing *S. aureus* and *P. aeruginosa*.

## 1. Introduction

Diabetic foot ulcer (DFU) is the most common manifestation of diabetes [1,2]. Treatments available for DFUs include debridement of wound necrotic tissues, wound dressings, administration of systemic and topical antimicrobial agents, and amputation as the last treatment option [3,4]. The number of diabetic patients undergoing major foot amputations has increased in recent years [5,6]. Besides, after a lower extremity amputation, half of the patients die or lose the contralateral limb within five years [2,7].

Infections in DFUs are the primary cause of lower-extremity amputation, and although most infections remain superficial, approximately 25% will spread from the skin to deeper subcutaneous tissues and bone [8,9]. Treatment of DFU infections are particularly challenging due to the presence of comorbidities, poor vascularization (determining reduced drug distribution in the lesional area), and microbial cell growth within a biofilm [10,11,12,13]. Indeed, biofilm acts as an important predisposing factor to the chronicity of a nonhealing ulcer by providing a protective environment against phagocytosis and decreasing the diffusion of antibiotics and antimicrobial agents [14,15,16,17,18]. Besides, previous studies have shown that the biofilm lifestyle promotes the horizontal transfer of virulence genes and the development of multidrug-resistant (MDR) organisms [19,20,21,22,23].

Directly related to the ability to form biofilms is the issue of antimicrobial tolerance [24,25]. Antibiotic tolerance is not mediated by the acquisition of antibiotic resistance genes [26] but rather is a transient phenotype that renders the bacterial cells within a biofilm highly refractory to antimicrobial therapy, allowing a subpopulation of cells to persist in the wound environment [27]. Despite the growing interest, biofilm production is not routinely analyzed in clinical microbiology testing [28,29,30,31]. Thus, considering the refractory nature of biofilm, the clinical management of infections caused by biofilm-growing bacteria, such those generally found in DFUs, requires the introduction of more targeted antimicrobial strategies [32,33].

Silver has a broad-spectrum antimicrobial activity against multidrug-susceptible and multidrug-resistant strains like *Pseudomonas aeruginosa*, extended-spectrum beta-lactamase (ESBL)-producing *Escherichia coli*, methicillin-resistant *Staphylococcus aureus* (MRSA), and vancomycin-resistant *S. aureus* (VRSA) [34,35,36,37,38]. Silver is toxic to microorganisms, affecting respiratory enzymes and components of the microbial electron transport system [39,40,41]. Besides, silver ions exert a bactericidal mechanism by binding to bacterial DNA and interfering with the transcription and replication processes [39,42]. Different formulations containing silver have been shown to eradicate bacterial biofilm in burns and slow-healing wounds [29,43,44,45,46]. Specifically, silver sulfadiazine (SSD) has proved to be an effective alternative to conventional antimicrobials, mainly when used topically in high concentrations directly at the site of infection [29,44,46].

The present study was aimed at evaluating the use of SSD for wound healing and infection control in patients with chronic DFUs infected with *S. aureus* and *P. aeruginosa* biofilms.

## 2. Experimental Section

### 2.1. Ethics, Patients, and Samples

The Central Ethics Committee I.R.C.C.S Lazio granted ethical approval for this study (Prot. CE/1016/15—15 December 2015, trials registry N. 730/15). Adult patients (9 females and 7 males; average age 63) with type 2 diabetes mellitus, all under insulin therapy, according to individual regimens, with a chronic DFU (>3 months) infected by *S. aureus* or *P. aeruginosa*, were recruited over a period between January 2017 and December 2019 at the Department of Plastic and Reconstructive Surgery, University of Rome “Tor Vergata”, and the IRCCS San Gallicano Dermatological Institute of Rome, Italy. Certain cases were complicated by diabetic nephropathy, retinopathy renal failure, and cardiovascular disease. The presence of the infection was defined according to specified guidelines [47]. All patients underwent vascular evaluation with Doppler vascular ultrasound in the absence of an indication of immediate surgical intervention. The exclusion criteria were as follows: patients diagnosed for cancer, patients who required vascular surgery at the time of admission or had received vascular surgery < 6 month before hospital admission, patient who had received immunosuppressive therapy, systemic or topical antimicrobial therapy 2 weeks before enrolment, patients who were discharged early or discontinued the therapy, were excluded from the study. One swab per patient was collected aseptically at baseline (T0—untreated patient) and after 30 days of treatment. Swabs were immediately transported to the microbiology laboratory and processed within 2 hours from collection [47].

All patients were treated with the complex dressing operative protocol in preparation for toilet surgery performed after 30 days of treatment. The protocol included disinfection; cleansing; application of SSD 1%; and application of nonadherent gauze, sterile gauze, and tibio-breech bandage. The dressing was changed every 72 h.

During the treatment period, different parameters including nonviable tissue (T), infection and/or inflammation (I), moisture imbalance (M), and nonadvancing edge of wound (E) were evaluated, according to the T.I.M.E. protocol [48,49,50], which allows for a systematic review of the characteristics of the lesion.

We used a modified T.I.M.E. protocol aimed at identifying some objective changes in tissue regeneration within each criterion and assigning them a score to classify the improvement to allow for a statistical assessment. Criterion T (bottom of the lesion): (1) presence of necrotic areas, (2) fibrinous bottom, (3) granulating bottom, (4) fund reduction with initial re-epithelialization. Criterion I (presence of clinically evident infection and inflammation): (1) present, (2) absent. Criterion M (moisture balance): (1) absent, (2) low, (3) medium, (4) high. Criterion E (edge of wound): (1) hyperkeratotic, (2) excoriated, (3) macerated, (4) undermined, (5) integrate.

### 2.2. Histological Evaluation

Incisional punch biopsies of ulcers (3 mm in diameter) were obtained at baseline (pretreatment, T0) and after 7 and 30 days of treatment with SSD. Microscopic evaluation of routinely hematoxylin–eosin-stained paraffin sections was performed to verify the healing process, and images were acquired using a digital camera (E600 Eclipse, Nikon, Tokyo, Japan).

### 2.3. Microbiological Assessment

Specimen collection and bacterial isolation from DFUs were performed as previously described [14]. *S. aureus* or *P. aeruginosa* were identified by the automatic VITEK 2 system (bioMérieux, Marcy-l’Étoile, France) and by matrix-assisted laser desorption/ionization time-of-flight mass spectrometry (MALDI-TOF, Bruker Daltonics, Bremen, Germany) [14,51].

### 2.4. Biofilm Production

*S. aureus* or *P. aeruginosa* strains, isolated from DFUs, were analyzed for their ability to produce biofilm by the clinical BioFilm Ring Test (cBRT) (Biofilm Control, Saint Beauzire, France) as previously described [31]. Each strain was analyzed in duplicate, and experiments were repeated three times.

### 2.5. Antimicrobial Susceptibility of Planktonic- and Biofilm-Grown Strains

The antimicrobial susceptibility testing (AST) was performed by the broth microdilution test (Thermo Scientific, Waltham, MA, USA) for the definition of the minimum inhibitory concentration (MIC) criteria, according to the European Committee on Antimicrobial Susceptibility Testing (EUCAST Clinical Breakpoint Table v 10.0). Biofilms of *S. aureus* or *P. aeruginosa* were grown in 96-well plates (Corning Inc., Corning, NY, USA) for the definition of the minimal biofilm-eradication concentration (MBEC) [25,52]. Results were interpreted according to the EUCAST Clinical Breakpoint (Table v 10.0). Briefly, wells were inoculated with approximately 1 × 10^5^ cells in 100 μL of BHI medium and incubated for 22 h at 37 °C to allow biofilm formation. Subsequently, the medium was removed, and the wells were washed with 100 μL of sterile distilled water to remove nonadherent cells. The preformed biofilms were treated with different antibiotics and SSD at predefined concentrations in fresh BHI medium and incubated for 22 h at 37 °C.

The metabolic activity of the treated planktonic and biofilm cultures was evaluated using the CellTiter-Blue staining (Promega Corporation, Madison, WI, USA) [53,54,55]. After 60 min of incubation, resorufin production was measured with the plate reader PhD lx System (Bio-Rad Laboratories, Hercules, CA, USA) using an excitation peak wavelength of 550 nm and an emission wavelength of 620 nm. Controls were carried out by replacing the culture medium with fresh BHI without SSD, and wells with the noninoculated medium were used as blanks. Percent resazurin reduction was calculated using the following formula: (experimental well absorbance – negative control absorbance)/positive control absorbance) × 100.

Tolerance factor (TF) calculation for SSD was adapted from Stewart [56] and measured according to the following equation:

TF = (RF_Sa_/RF_Pa_) (1)

where RF refers to a calculated reduction of relative fluorescence according to the resazurin viability assays for *S. aureus* (Sa) and *P. aeruginosa* (Pa) at different concentrations of SSD.

Measurements were performed in triplicate for at least three independent experiments, and the results are expressed as mean and standard deviation.

### 2.6. Biofilm Imaging

Biofilms were grown in μ-Slide (Ibidi, Gräfelfing, Germany) inoculated with ~1 × 10^5^ cells in 500 μL of fresh BHI medium and incubated for 48 h at 37 °C. The culture medium was changed after 24 h of biofilm growth. Then, biofilms were treated with different concentrations of SSD in fresh BHI medium and incubated for an additional 22 h at 37 °C. Biofilms were stained using the LIVE/DEAD BacLight Bacterial Viability Kit (Life Technologies, New York, NY, USA), according to supplier specifications and examined with Apotome Fluorescence Microscope (Carl Zeiss International, Oberkochen, Germany). Data were analyzed with the AxioVision 4.8 software [57].

### 2.7. Statistical Analysis

Statistical analysis was performed using the chi-square test when applicable. The Wilcoxon test was used to compare the distributions of cases at T0 and T1, and the Mann–Whitney U-test was used for comparing the features of patients with *S. aureus* with those of patients with *P. aeruginosa*. Statistical analyses were carried out using IBM SPSS v.21 statistics software. Differences were considered statistically significant for values of *p* < 0.05 (*), *p* < 0.01 (**), and *p* < 0.001 (***).

## 3. Results

A total of 16 participants with infected DFUs and matching the study inclusion criteria were enrolled within the 2017–2019 timeframe. The study group included 7 (44%) males and 9 (56%) females with a mean age of 63.6 years (SD: ±13.7). The most common location for DFUs was on the forefoot area (N6; 37.4%), followed by the heel (N4; 25%), the mid-foot area (N3; 18.8%), and the toe tips (N3; 18.8%). Ulcer characteristics, according to the T.I.M.E. classification system, are summarized in Table 1. All the study participants were treated with SSD after careful disinfection and cleansing of the wound bed and application of nonadherent gauze, sterile gauze, and tibio-breech bandage every 72 h.

**Figure 1:**
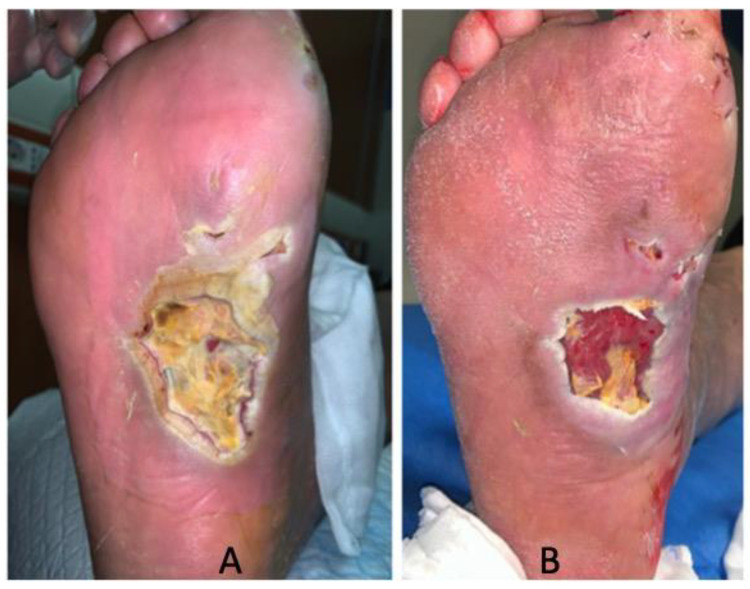
DFU healing. (**A**) Wound at baseline (T0) and (**B**) after 30 days of treatment.

Data showed a statistically significant improvement of patients’ ulcers after 30 days of treatment according to the T.I.M.E. (Table 1). The wound healing and the decrease in wound size are also shown in Figure 1. Notably, the resolution of infection was observed after 30 days of treatment with SSD in all the DFUs. Furthermore, no significant differences were found between patients with DFUs infected by *S. aureus* and *P. aeruginosa* at T0 in terms of tissue (*p* = 0.264), infection (*p* = 0.999), exudate (*p* = 0.777), and the edge of the wound (*p* = 0.375). Similarly, no significant differences were found after 30 days among patients for tissue (*p* = 0.288), infection (*p* = 0.999), exudate (*p* = 0.814), and the edge of the wound (*p* = 0.535). No adverse events related to the SSD treatment were reported during the study period. A representative image of wound healing and decrease in wound size is shown in Figure 1.

### 3.1. Microscopic Evaluation

**Figure 2:**
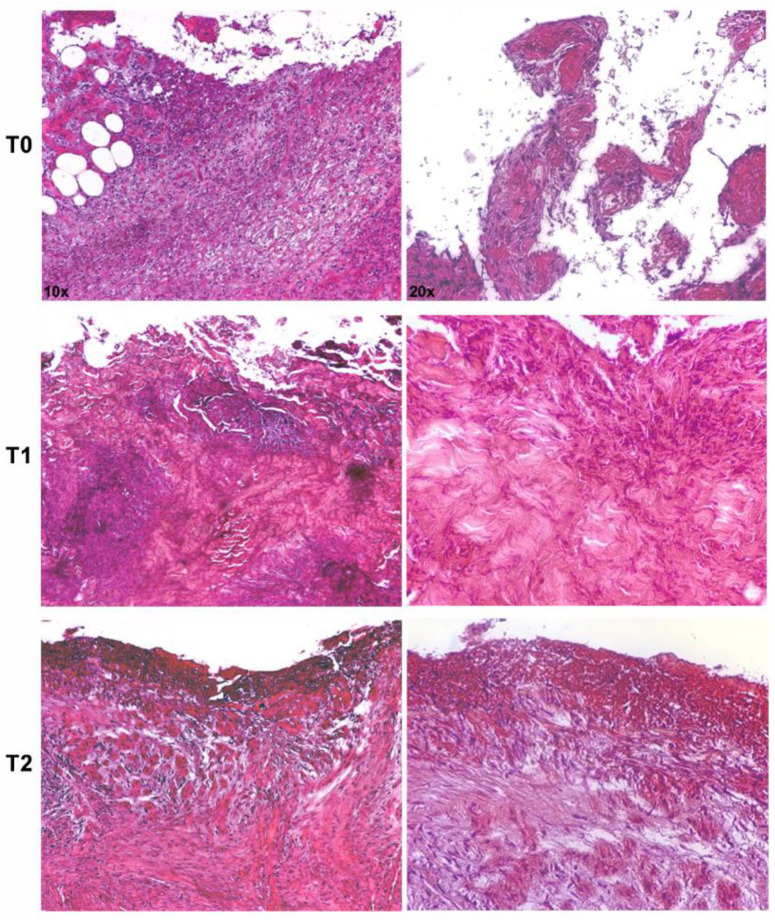
Representative microphotographs of hematoxylin–eosin-stained paraffin sections of skin biopsies show progressive healing of an infected ulcer treated with silver sulfadiazine (SSD). The pretreatment biopsy (**T0**) presented an intense inflammatory infiltrate in the dermis and evident clusters of microbial agents along the epithelial borderline. After 7 (**T1**) and 30 days (**T2**) of treatment, a significant reduction of the inflammatory infiltrate and microbial agents were observed, with the deposition of new collagen (magnification: 10× and 20×).

Representative microphotographs of hematoxylin–eosin staining, describing the evolution of the healing process of an infected DFU, are shown in Figure 2. An evident healing process was documented after 7 and 30 days of treatment as compared with the pretreatment biopsy, which showed an intense inflammatory infiltrate, cellular debris, and edema. Microbial clusters were visible along the epithelial borderline. After treatment, the healing process was accompanied by a reduction of the inflammatory infiltrate and microbial agents and an increase of newly formed dermal tissue.

### 3.2. Biofilm Production

**Figure 3:**
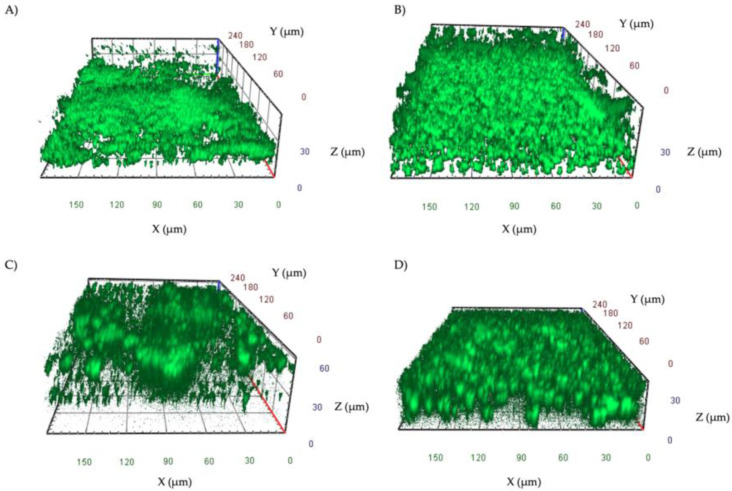
Representative images of biofilms of *S. aureus* (**A**,**B**) and *P. aeruginosa* (**C**,**D**) isolates grown in IBIDI μ-slides for 48 h at 37 °C. Orthogonal sections displaying horizontal (z) and side views (x and y) of reconstructed 3D biofilm images are shown.

A total of 16 strains, including eight *S. aureus* and eight *P. aeruginosa*, isolated from patients with DFUs, were analyzed. All the isolates were found to be strong biofilm-producers by the cBRT. Confocal microscopy analysis of the biofilms (Figure 3) was performed after 48 h of incubation to develop a mature biofilm. *S. aureus* isolates (Figure 3A,B) formed a uniform layer of biofilm of 25–40 μm. *P. aeruginosa* isolates (Figure 3C,D) formed either pronounced mushroom-shaped structures of 40–60 μm or a uniform layer of cells of 25–40 μm.

### 3.3. Antimicrobial Susceptibility Testing of Planktonic and Biofilm Cells

The antibiotic susceptibility profiles (AST) of *S. aureus* and *P. aeruginosa* isolates, determined according to EUCAST breakpoint guidelines, are summarized in Table 2. All the *S. aureus* isolates were found susceptible to daptomycin (MIC 0.25 to 1 mg/L), fusidic acid (MIC ≤0.5 mg/L), gentamicin (MIC ≤0.5 mg/L), linezolid (MIC 2 mg/L), teicoplanin (MIC ≤0.12 mg/L), tigecycline (MIC ≤0.12 to 0.25 mg/L), trimethoprim/sulfamethoxazole (TMP/SMX) (MIC ≤0.25 to 1 mg/L), and vancomycin (MIC ≤0.5 to 1 mg/L). Besides, seven (87.5%) *S. aureus* isolates were susceptible to oxacillin (MIC ≤0.25 mg/L), six (75%) to clindamycin (MIC ≤0.25 mg/L), five to erythromycin (MIC ≤0.25 to 1 mg/L), and one to benzylpenicillin (MIC 0.25 mg/L). Only one strain was classified as MRSA with an oxacillin MIC of 4. Notably, all *P. aeruginosa* isolates were susceptible to colistin (MIC ≤0.25 mg/L) and piperacillin/tazobactam (PIT) (MIC ≤4 to 8 mg/L), while seven (87.5%) isolates were susceptible to imipenem (MIC 0.25 to 1 mg/L) and six (75%) isolates were susceptible to amikacin (MIC ≤2 mg/L), cefepime (MIC ≤1 mg/L), ceftazidime (MIC 2 to 4 mg/L), ciprofloxacin (MIC ≤0.25 to 0.5 mg/L), and gentamicin (MIC ≤ 1 to 2 mg/L). Only one strain was classified as MDR, resulting as susceptible only to colistin (MIC ≤0.25 mg/L) and PIT (MIC ≤4 mg/L).

The antibiotic susceptibility of *S. aureus* and *P. aeruginosa* isolates in biofilm significantly (*p* < 0.001) differed from those gathered by AST (Table 1). In particular, of the *S. aureus* isolates analyzed in biofilm, two (25.0%) were susceptible to fusidic acid (MBEC 0.5 mg/mL), oxacillin (MBEC 0.25 to 1 mg/L), and teicoplanin (MBEC 1 mg/L), but all the isolates were found to be resistant to benzylpenicillin (MBEC >8 mg/L), TMP/SMX (MBEC >4 mg/L), and vancomycin (MBEC >4 mg/L). The *P. aeruginosa* isolates in the biofilm were found susceptible in two cases to amikacin (MBEC 4 mg/L) and gentamicin (MBEC 4 mg/L). Notably, all isolates were resistant to cefepime (MBEC >32 mg/L), ceftazidime (MBEC ≥32 mg/L), ciprofloxacin (MBEC 1 to >2 mg/L), and PIT (MBEC >128 mg/L).

### 3.4. Silver Sulfadiazine Susceptibility Testing of Planktonic and Biofilm Cells

The *S. aureus* and *P. aeruginosa* isolates in biofilm exhibited a considerable increase of antibiotic tolerance as compared to their planktonic counterparts. SSD has been suggested as an effective alternative for local treatment of wound infections, even against biofilm [43,48,58]. In this study, the SSD activity was initially evaluated by the broth microdilution test on planktonic isolates. Notably, all the *S. aureus* and *P. aeruginosa* isolates showed MIC ≤0.16 mg/L. The antimicrobial activity of SSD was further assessed in biofilm-growing cells. *S. aureus* isolates exhibited MBEC ranging from 1.25 to 2.5 mg/L, while *P. aeruginosa* showed MBEC values ranging from 0.16 to 0.31 mg/L. The results are summarized in Figure 3.

**Figure 4:**
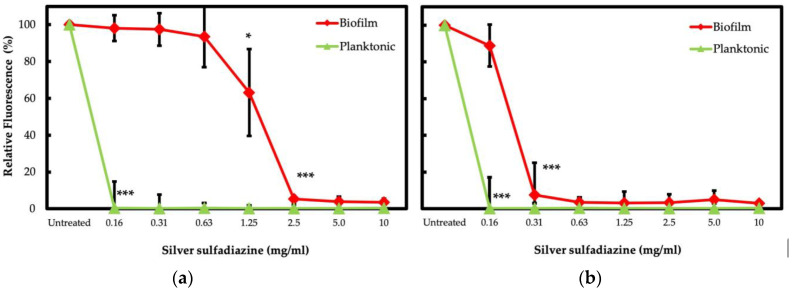
Viability of bacterial isolates treated with SSD. *S. aureus* (**a**) and *P. aeruginosa* (**b**) were exposed to different concentrations of SSD for 24 h. Bacterial cells were incubated with the BHI medium in the presence of resazurin. The resorufin production was quantified by measuring fluorescence (relative fluorescence), relative to the untreated control, after 60 min of incubation for *S. aureus* and *P. aeruginosa*. *p* < 0.05 (*) and *p* < 0.001 (***).

Planktonic cells of *S. aureus* and *P. aeruginosa* isolates exhibited a significant (*p* < 0.001) decrease in cell viability at 0.16 mg/mL of SSD when compared to the untreated controls. The viability of *S. aureus* in biofilm was significantly (*p* = 0.01) reduced by 36.8% in the presence of 1.25 mg/mL of SSD and experienced a severe (*p* < 0.001) reduction of 94.7% when exposed to a concentration of 2.5 mg/mL (Figure 4a). Notably, the resazurin assay showed that the viability of *P. aeruginosa* isolates was significantly (*p* < 0.001) reduced by 92.6% when biofilms were exposed to a concentration of 0.31 mg/mL of SSD (Figure 4b).

The rate of killing by SSD was lower for *S. aureus* than *P. aeruginosa* (Table 3). This analysis was performed by adapting the calculation of tolerance factor (TF) described by Stewart [56]. Biofilm-growing *S. aureus* and *P. aeruginosa* isolates were found to be equally tolerant to SSD at 0.16 mg/mL. Conversely, *S. aureus* resulted in 26.7 and 20.0 times more tolerant than *P. aeruginosa* at 0.63 and 1.25 mg/mL. However, *S. aureus* and *P. aeruginosa* were both found highly susceptible at concentrations of SSD between 2.5 and 10 mg/mL.

## 4. Discussion

DFU is a severe and frequent complication of diabetes mellitus worldwide and the most common cause of hospitalization in diabetic patients. Approximately half of DFUs become infected [8], and amputation is required in more than 15% of cases [9,59]. Systemic antibiotics are prescribed in the presence of clinical signs of DFU infection [60,61,62]. However, the resolution of infection after antibiotic treatment varies widely, with values ranging between 5.6% and 77.8% [63].

The presence of a microbial biofilm within the host tissue poses a significant clinical complication. Biofilm-associated infections exhibit high resistance to host defenses, often contributing to an excessive or inappropriate inflammatory response that, in turn, leads to further tissue damage and spreading of the infection [64,65]. The present study was aimed at assessing the use of SSD on DFUs infected by biofilm-producing *S. aureus* and *P. aeruginosa* in 16 diabetic patients. Clinical data showed that the application SSD after careful disinfection and cleansing of the wound bed allowed a significant reduction of the exudate and local infection signs, along with the preservation of the structural and anatomical characteristics of the treated areas. Notably, after 30 days of treatment, wound sampling gave negative microbial cultures in all patients, suggesting that SSD may represent a useful prophylactic and a broad-spectrum antimicrobial agent [35,36,37,40]. The histological images showed that the presurgery period was characterized by the presence of an intense inflammatory infiltrate, cellular debris, and edema in the dermis with evident groups of microbial aggregates along the epithelial border. After about two weeks of treatment, a reduction in the inflammatory infiltrate and microbial aggregates was observed, along with the deposition of new collagen. These results confirmed that the beneficial effects of the SSD on wound management are mostly correlated to both antimicrobial and anti-inflammatory activity [38].

The conventional AST revealed that the *S. aureus* and *P. aeruginosa* isolates were highly susceptible to most antibiotics tested. Specifically, *S. aureus* was found highly susceptible to daptomycin, fusidic acid, gentamicin, linezolid, teicoplanin, tigecycline, TMP/SMX, and vancomycin, with MIC values comparable to previous studies [66,67,68,69]. Notably, the MIC value for methicillin-susceptible *S. aureus* (MSSA) was ≤0.25 mg/L, and one strain was found resistant to oxacillin with MIC ≥4 mg/L. The increased prevalence of MRSA in DFUs has promoted a return to non-β-lactam antimicrobial agents, such as rifampicin, fusidic acid, and TMP/SMX [68]. Although these agents have proven to be effective in treating DFUs, they are believed to enhance antibiotic resistance [2]. Besides, dalbavancin showed good antimicrobial activity in diabetic foot infections, showing higher activity than vancomycin, daptomycin, and linezolid against MRSA and MSSA [66,70].

Colistin and PIT were found to be the most effective drugs against *P. aeruginosa* isolate, with MIC ≤0.25 mg/L and ≤4 to 8 mg/L, respectively. Imipenem was active against 87.5% of strains. Besides, 75% of *P. aeruginosa* isolates were susceptible to cefepime and ceftazidime. These findings are also consistent with the results reported by other studies from different countries showing that colistin, β-lactams, and aminoglycosides were effective against *P. aeruginosa* isolated from patients with diabetic foot infection [14,69,71,72]. Likewise, aminoglycosides and ciprofloxacin were effective against 75% of the isolates. A microbiological survey conducted in Italy on patients with DFUs showed comparable results in antibiotic susceptibility rates. Specifically, colistin was found to be the most effective antibiotic, with a susceptibility rate above 90% against *P. aeruginosa* isolates [68]. Besides, approximately 80% of *P. aeruginosa* strains were found to be susceptible to PIT, cefepime, and ceftazidime [68]. Previous studies also reported a comparable trend for PIT on *P. aeruginosa* isolates from DFUs [72,73,74]. Aminoglycosides were found effective in more than 74% of cases [68]. However, studies from Pakistan found that *P. aeruginosa* was more susceptible to quinolones but less susceptible to β-lactams [69,75]. Ciprofloxacin was considered to be effective against *P. aeruginosa* infections [74]. However, more recent studies showed that approximately 50% of *P. aeruginosa* isolates from diabetic wounds were resistant to this antibiotic [72]. This result may reflect substantial geographical variations in the use of antibiotics and antibiotic prescriptions.

The extraordinary tolerance to antimicrobial agents observed in vivo in patients with chronic DFU infections is apparently in contrast with the AST profiles gathered in this study. Biofilm formation by pathogenic bacteria is a characteristic hallmark of chronic DFU infections [76,77,78]. Biofilm has been reported in 77% of patients with DFUs, and biofilm-embedded cells were found to be more tolerant to antibiotic treatments than planktonic cells. Thus, antibiotic treatments based on planktonic cells’ susceptibility profiles may lead to recurrent and difficult-to-treat wound infections [29,79,80]. Despite its recognized importance, biofilm is not assessed in chronic wound infection, and the detection of the biofilm remains a difficult task in routine clinical laboratories.

The cBRT and confocal microscopy analysis showed that all the clinical isolates analyzed in this study developed complex, three-dimensional biofilm structures. Notably, the confocal microscopy images were highly consistent with the results obtained by the cBRT, revealing a biofilm matrix between 25 and 60 μm in height with all isolates (Figure 2). This result is in accordance with previous studies showing a high level of biofilm production for *S. aureus* and *P. aeruginosa* isolated from chronic DFUs [31,76,81].

The antibiotic concentration required to eradicate biofilm bacteria can be several orders of magnitude higher than that required for the same microorganism in the planktonic state [25,82,83,84]. The antibiotic susceptibility profiles do not consider the presence of biofilm-growing microorganisms and might not represent the bacterial drug susceptibility in vivo [85]. Thus, using the criteria recommended by the EUCAST for the determination of MIC, we evaluated the antimicrobial susceptibility profile of the biofilm-growing isolates. In this study, biofilm cultures of *S. aureus* and *P. aeruginosa* were found to be significantly (*p* < 0.001) more tolerant than their planktonic counterparts to all antibiotics tested. Specifically, *S. aureus* isolates were found to be fully tolerant to vancomycin and TMP/SMX when assessed in a biofilm. Fusidic acid, oxacillin, and teicoplanin were the most active drugs against *S. aureus* biofilm. However, MBEC values remained below breakpoints in only 25% of cases. The most effective antibiotics against and *P. aeruginosa* biofilm were amikacin and gentamicin, with MBEC values below the breakpoints in 25% of cases. The efficacy of gentamicin was previously demonstrated against *S. aureus* and *P. aeruginosa* biofilm in an in vitro model studying the effectiveness of different treatments for infected DFUs. However, this study concluded that gentamicin was active against biofilm as a topical antibiotic but inadequate when administered systemically [86]. After systemic administration, the wound’s antibiotic concentration is lower than that detected in the serum at any given time [87]. A previous study reported that vancomycin penetration into soft tissue is reduced in diabetic patients [88]. Besides, other reports have described a variable penetration of antibiotics into the soft tissue of diabetic patients [86,89,90,91]. Peripheral artery disease (PAD) is a significant risk factor in chronic wounds caused by reduced blood flow and immune involvement at the site of infection [92,93]. Studies of antibiotic concentrations in DFIs generally do not include patients with PAD; thus, the concentration of antibiotics reaching tissue may be even lower than reported [13]. The reduced penetration of antibiotics in the site of infection may offer selective pressure to promote antibiotic resistance [94,95]. Thus, in the presence of high-risk infected DFUs, it has been proposed that topical antimicrobial therapy may represent a more appropriate option to reduce bacterial bioburden and accelerate healing [96]. Local administration of SSD is considered effective in treating infected wounds [43,58,97]. Our data confirm the efficacy of SSD against planktonic *S. aureus* and *P. aeruginosa* isolates at MIC of ≤0.16 mg/L. Previous in vitro tests have demonstrated a strong antibacterial activity of SSD against *S. aureus* and *P. aeruginosa* strains at concentrations lower than those generally used in clinical preparations (10 mg/mL) [98]. The antimicrobial activity of SSD was further assessed in biofilm-growing cells. *S. aureus* isolates exhibited MBEC ranging from 1.25 to 2.5 mg/L, while *P. aeruginosa* showed MBEC values ranging from 0.16 to 0.31 mg/L. Previous works have proved that SSD is effective at eliminating *S. aureus* and *P. aeruginosa* biofilms at concentrations below 10 mg/mL [48,99,100]. Besides, it has also been observed that SSD concentrations between 5 and 10 mg/mL are effective against mature biofilms of *P. aeruginosa* [58]. The rate of killing by SSD was lower for *S. aureus* than *P. aeruginosa*. Biofilm-growing *S. aureus* and *P. aeruginosa* isolates were found to be equally tolerant to SSD at 0.16 mg/mL. Notably, *S. aureus* resulted in 26.7 and 20.0 times more tolerant than *P. aeruginosa* at 0.63 and 1.25 mg/mL.

Most studies have demonstrated that SSD is nontoxic. However, the overuse of SSD and silver derivatives can accumulate in the skin, causing skin irritation and argyria. Allergic contact dermatitis to SSD has been reported, although most of the toxic effects were related to the excipients. After absorption, silver has been found in different tissues, including the liver, kidney, heart, brain, eye, and other organs. Burn patients treated with SSD cream showed elevated serum silver (over 20 mg L^−1^). However, this occurred after prolonged exposure of leg ulcers and acute burns to 1% SSD [101]. Different in vitro studies have also described the concentration-dependent toxicity of silver in mammalian cell lines such as keratinocytes or fibroblasts [101]. This evidence suggests that the judicious use of silver-containing dressings is essential to limit toxicity and optimize wound healing.

A potential limitation of this study is the use of monocultures of *S. aureus* or *P. aeruginosa* that may not reflect the polymicrobial nature of most DFU infections [2,18,102,103,104]. Indeed, a polymicrobial population, particularly when embedded in a biofilm, may have a more structured biofilm and a significantly increased tolerance to antibiotics [56,105,106,107,108]. Besides, our study would also benefit from validation in a larger cohort of patients and from studying other types of bacteria in mono- or polymicrobial cultures. The diversity of the bacterial populations in DFUs is considered an important contributor to the chronicity of the ulcers [2,18,103,104]. However, in patients with chronic DFUs and under antibiotic therapy, like those enrolled in this study, monomicrobial infection is common. *S. aureus* and *P. aeruginosa* represent the most prevalent and clinically relevant pathogens associated with severe or even fatal infections [47,104,109,110,111,112,113]. Taken together, the findings presented in this study may provide relevant information for avoiding unnecessary or prolonged antibiotic therapy and addressing an appropriate targeting of therapeutic intervention in chronic DFUs.

## 5. Conclusions

The therapeutic protocol presented in this study was based on topical SSD application in preparation for the surgical toilet. Results showed a significant improvement after 30 days of treatment in all the T.I.M.E. parameters with a reduction of the local infection signs and optimal infection control. Besides, this study revealed that *S. aureus* and *P. aeruginosa* isolated from infected DFUs developed complex and highly structured biofilms in vitro. The choice of antibiotic therapy is generally based on the causative pathogens. However, in the presence of highly tolerant biofilm-growing bacteria, the antibiotic susceptibility profiles might not be representative of the bacterial drug susceptibility in vivo. SSD was found to be effective against fully developed biofilms of both *S. aureus* and *P. aeruginosa* at concentrations below those normally used in clinical preparations (10 mg/mL). The recognized importance of the microbial biofilm in chronic wound infections has led to the proposal of biofilm-based wound care (BBWC) [80]. This clinical guideline suggests a combination of treatment with a broad-spectrum antibiotic and application of a local antibiofilm agent accompanied by sharp debridement of the wound [80]. Our results further support the introduction of a BBWC protocol and provide the basis for the clinical validation of a novel diagnostic approach aimed at defining biofilm-specific eradication strategies for the management of chronic DFU infections in a personalized manner.

## Figures and Tables

**Table 1:** Clinical characteristics of the diabetic foot ulcers (DFUs) reported as absolute frequencies and percentages (%) at baseline (T0) and 30 days. The distributions were compared for each variable using the Wilcoxon test, and the relevant *p*-values are reported.

Variable	Description	T0	30 Days	*p*-Value
Tissue	Areas of necrosis	4 (25%)	0 (0%)	<0.001
	Fibrin	12 (75%)	2 (12.5%)	
	Granulation	0 (0%)	9 (56.3%)	
	Epithelialization	0 (0%)	5 (31.2%)	
Infection	Present	16 (100%)	0 (0%)	<0.001
Exudate	Absent	2 (12.5%)	8 (50%)	<0.001
	Low	3 (18.7%)	7 (43.8%)	
	Medium	7 (43.8%)	1 (6.2%)	
	High	4 (25%)	0 (0%)	
Edge of wound	Hyperkeratotic	2 (12.5%)	0 (0%)	0.002
	Excoriated	3 (18.7%)	0 (0%)	
	Maceration	7 (43.8%)	3 (18.7%)	
	Undermining	4 (37.5%)	0 (0%)	
	Integrated	0 (0%)	13 (81.3%)	

**Table 2:** Antibiotic susceptibility profile (% of susceptible strains) of *S. aureus* and *P. aeruginosa* as obtained by the antimicrobial susceptibility testing (AST) and the anti-biofilm test (ABT). PIT, piperacillin/tazobactam; TMP/SMX, trimethoprim/sulfamethoxazole.

Drug	AST		ABT	
	*S. aureus*	*P. aeruginosa*	*S. aureus*	*P. aeruginosa*
Amikacin	-	75	-	25
Benzylpenicillin	12.5	-	0	-
Cefepime	-	75	-	0
Ceftazidime	-	75	-	0
Ciprofloxacin	-	75	-	0
Clindamycin	75	-	12.5	-
Colistin	-	100	-	12.5
Daptomycin	100	-	12.5	-
Erythromycin	62.5	-	12.5	-
Fusidic Acid	100	-	25	-
Gentamicin	100	75	12.5	25
Imipenem	-	87.5	-	12.5
Linezolid	100	-	12.5	-
Oxacillin	87.5	-	25	-
PIT	-	100	-	0
Teicoplanin	100	-	25	-
Tigecycline	100	-	12.5	-
TMP/SMX	100	-	0	-
Vancomycin	100	-	0	-

**Table 3:** Tolerance factor (TF) between *S. aureus* (Sa) and *P. aeruginosa* (Pa), calculated at different concentrations of SSD.

[SSD] (mg/mL)	TF (Sa/Pa)
0.16	1.1
0.31	13.0
0.63	26.7
1.25	20.0
2.5	1.6
5	0.8
10	1.2
